# Classification of the plant-associated lifestyle of* Pseudomonas* strains using genome properties and machine learning

**DOI:** 10.1038/s41598-022-14913-4

**Published:** 2022-06-27

**Authors:** Wasin Poncheewin, Anne D. van Diepeningen, Theo A. J. van der Lee, Maria Suarez-Diez, Peter J. Schaap

**Affiliations:** 1 Laboratory of Systems and Synthetic Biology, Wageningen University & Research, Wageningen, The Netherlands; 2 BU Biointeractions and Plant Health, Wageningen Plant Research, Wageningen University & Research, Wageningen, The Netherlands; 3 UNLOCK Large Scale Infrastructure for Microbial Communities, Wageningen University and Research, Wageningen, The Netherlands

**Keywords:** Classification and taxonomy, Data mining, Data processing, Functional clustering, Genome informatics, Machine learning, Bacterial genomics, Bacterial pathogenesis, Pathogens

## Abstract

The rhizosphere, the region of soil surrounding roots of plants, is colonized by a unique population of Plant Growth Promoting Rhizobacteria (PGPR). Many important PGPR as well as plant pathogens belong to the genus *Pseudomonas*. There is, however, uncertainty on the divide between beneficial and pathogenic strains as previously thought to be signifying genomic features have limited power to separate these strains. Here we used the Genome properties (GP) common biological pathways annotation system and Machine Learning (ML) to establish the relationship between the genome wide GP composition and the plant-associated lifestyle of 91 *Pseudomonas* strains isolated from the rhizosphere and the phyllosphere representing both plant-associated phenotypes. GP enrichment analysis, Random Forest model fitting and feature selection revealed 28 discriminating features. A test set of 75 new strains confirmed the importance of the selected features for classification. The results suggest that GP annotations provide a promising computational tool to better classify the plant-associated lifestyle.

## Introduction

Among the targets set by the United Nations to achieve the zero-hunger goal, the need to double the agricultural food production is specified^1^. Earlier attempts to improve plant performance and production focused on plant breeding, pest control by chemical means and the implementation of synthetic fertilizers tapping into finite global reserves^2,3^. While these strategies were successful in enhancing production, the increasing adverse effects on the environment challenges us to find sustainable alternatives^4–6^.

A multitude of studies has demonstrated that cooperative microbiomes can play important positive roles in plant growth, development, and fitness^2,3,7^. One particular hotspot is the rhizosphere, the region of soil surrounding plant roots, colonized by Plant Growth Promoting Rhizobacteria (PGPR)^8^. A stable PGPR population can increase the stress tolerance, growth and yield of crop plants by enhancing nutrient uptake from the soil and through modulation of plant phytohormone status and metabolism^7,9–15^. The most studied PGPR are *Pseudomonas* spp., a functionally diverse group representing plant beneficial as well as (opportunistic) pathogenic species such as *P. syringae* that can live on the plant surface as an epiphyte. Under right conditions *P. syringae* can also colonize the interior tissue of the plant and cause disease^16–18^.

The plant-associated lifestyle of a *Pseudomonas* strain is the result of a diverse spectrum of plant-host interaction pathways. Genome based correlational approaches have identified a number of marker genes contributing to the phenotype^19–21^. These marker genes are however, to a certain degree, shared between both groups^22^ and consequently, the uncertainty on the divide increases with each new genome added. Until now, a generic description of presence and completeness of biological functions and pathways contributing to the plant-associated lifestyle of a *Pseudomonas* strain is lacking. Such knowledge would bring fundamental insights into their potential to enhance plant performance and resilience.

Comparative functional genomics is possible when genes are placed in biological context. Genome Properties (GP) is domain-based functional annotation system whereby functional attributes can be assigned to a genome^23^. The resource represents a collection of 1286 common biological pathways evidenced by a distinct sets of protein domains. For a functional comparison at a larger scale, protein domains are better scalable and less sensitive to sequence variation compared to techniques based on sequence similarity ^24,25^. Here we applied GP-based functional genomics using the total of 1286 features and machine learning techniques to compare 91 completely sequenced *Pseudomonas* strains with a documented lifestyle: 58 soil-dwelling *Pseudomonas* strains classified as PGPR and 33 known plant-pathogens, mostly epiphytic *P. syringae* strains (EPP). As strains with different lifestyles often belong to a single species, it was suggested that genomic islands gained and lost through homologous recombination may encode important determinants of the plant-associated lifestyle^26^. A system wide analysis of the Genome Properties encoded by these variable regions allowed us to accurately classify *Pseudomonas* strains, and to identify new discriminating functional features that may contribute to the plant-associated lifestyle. In the discussion section these discriminating features are placed into a biological context.

## Results

**Figure 1:**
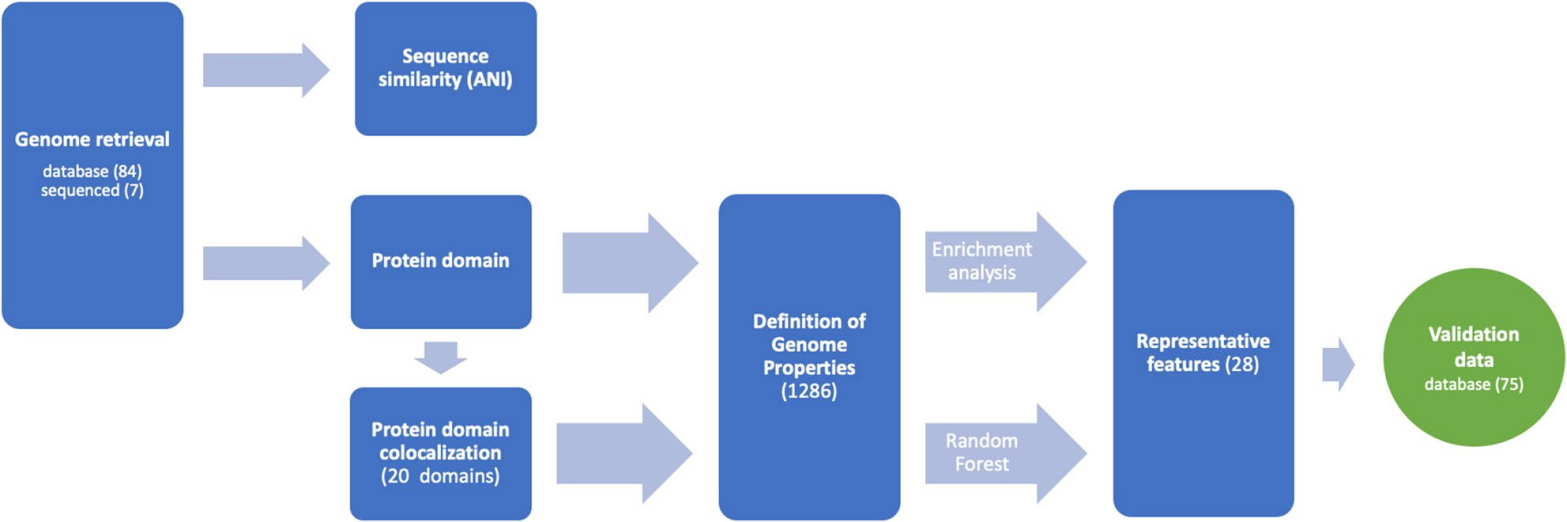
Workflow for GPs based functional genomics and classification. Genome sequences are analyzed using sequence similarity and protein domain content. (Colocalized) protein domain content is used to infer Genome Properties. Enrichment analysis and Random Forest feature selection was used obtain genomic features. Classification performance was evaluated using a test set of 75 newly available genomes.

Based on literature review, the complete genomes of 84 *Pseudomonas* strains were retrieved from the Pseudomonas Genome DB (version 17.2)^27^ and categorized as encoding either a ‘PGPR’ strain (51 strains) or a ‘EPP’ strain (33 strains) (see Supplementary Table S1 for details). This selection was supplemented with the complete genomes of seven new or re-sequenced PGPR strains; *P. putida* P9, *P. corrugata* IDV1, *P. fluorescens* R1 and WCS374, *P. protegens* Pf-5, *P. chlororaphis* Phz24 and *P. jessenii* RU47. To avoid gene and protein domain annotation inequality, all 91 strains were de novo annotated. Subsequently, the two groups were compared by nucleotide sequence similarity, by protein domain presence and by presence and completeness of domain-based GPs (Fig. 1). Domain content was subjected to enrichment analysis and the domain based GP content was used to train and validate a Random Forest (RF) model for classification purposes and feature selection^28^. The performance of the classification methods was tested using a set of 75 plant associated *Pseudomonas* genomes obtained from a newer version (V20.2) of the Pseudomonas Genome DB.

### Sequence similarity

**Figure 2:**
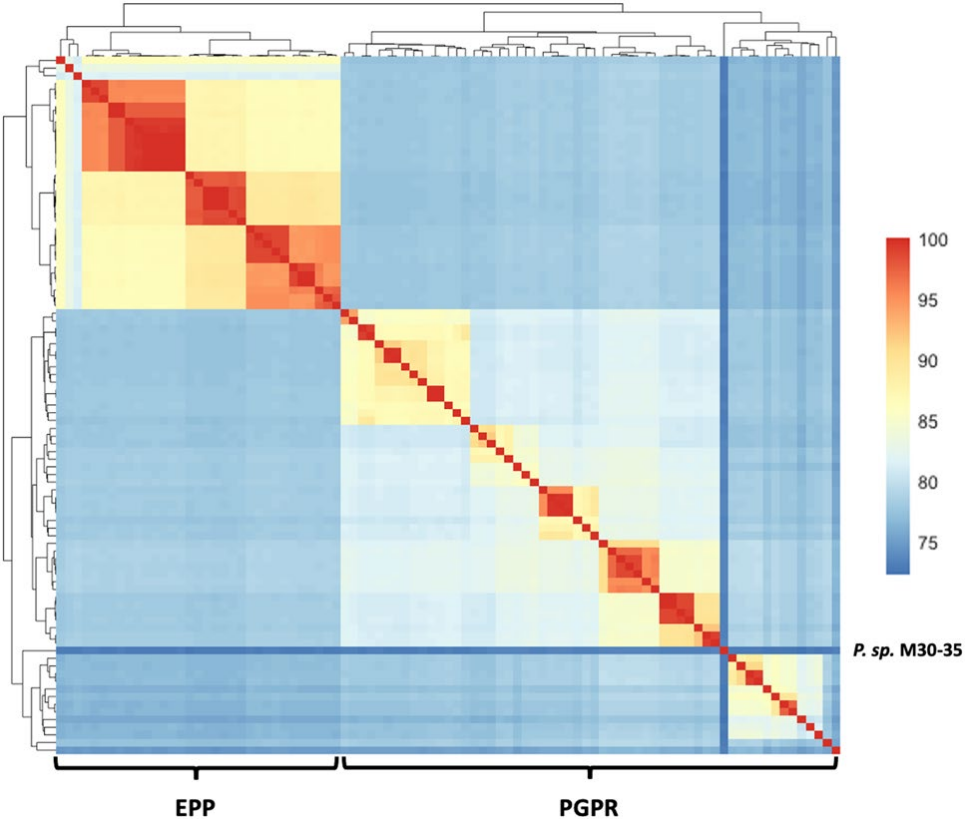
Pairwise Average Nucleotide Identity (ANI) scores between coding regions. Scores were calculated from alignments that have 70% or more identity and at least 70% coverage of the shorter gene.

We first examined the global genomic relatedness between the PGPR and EPP group, by calculating the Average Nucleotide Identity (ANI) scores between all possible pairs (Fig. 2). The ANI scores showed that corresponding with their phenotypic classification the genome sequences could be divided into two groups with *Pseudomonas sp.* M30-35 being less similar to the rest of the PGPR group. The average sequence similarity within the PGPR and EPP group was 79.57 ± 4.27 and 90.01 ± 5.53, respectively. The ANI-score measures the global similarity between the coding regions of two genomes at nucleotide-level taking into account hits that have 70% or more identity and at least 70% coverage of the shorter gene. The ANI score does not consider the fraction of coding sequences that contribute to this score and thus provides no insight in strain-specific functional adaptations. To study the impact of strain-specific functional adaptations, the protein domain content of each strain was considered.

### Protein domain content

The 91 de novo annotated complete *Pseudomonas* genomes on average code for 5640 ± 643 protein encoding genes. As many proteins consist of multiple domains, for each genome, 9342 ± 709 domains could be identified with an average domain copy number of 2.35 ± 0.12 (Supplementary Table S1).

Using domain presence/absence as input, a group-wise enrichment analysis was done and a total of 410 and 329 protein domains were found to be significantly enriched in respectively PGPR and EPP strains (Supplementary Table S2). PGPR strains were enriched for five domains linked to Type II secretion systems (T2SS), ten domains linked to the term “cytochrome”, eight domains linked to, “quinohemoprotein” and six domains linked to “biofilm” (Poly-beta-1,6-N-acetyl-D-glucosamine type) biosynthesis. Interestingly, domains related to “quinohemoprotein” and “biofilm” were not only enriched but also exclusively found in PGPR strains. EPP strains were enriched with domains involved in various types of other secretion systems. Moreover, some of these domains were not present in any of the PGPR strains_._ Eighteen of those in EPP enriched domains are reported to be involved in the Type III secretion system and five in the Type IV secretion system. In addition, the EPP list showed enrichment of nine different domain involved in phosphonate metabolism. Shared synteny and functional clustering of enriched domains was further explored using genome properties.

### Genome properties

Genome properties (GP) represent a collection of currently 1286 common biological pathways. Each GP consists of a precomputed cluster of essential core protein domains which are used as evidences for the presence of the biological pathway^23^. Genome derived protein domains were used to construct for each strain a list of GPs with two possible evidence values: ‘COMPLETE’ indicating that the complete set of precomputed evidences had been detected and ‘PARTIAL’, indicating a likely presence of the corresponding GP due to the presence of an incomplete set of evidences above a per GP specified minimal threshold. In addition, we considered that the bacterial genes encoding domains that function in the same biological pathway are often arranged in operonic structures corresponding to syntenic blocks. For each strain GPs were therefore reconstructed not only based on protein domain presence (GP-PA) but also on protein domain colocalization (GP-SND; non-directional) and on domain colocalization and being encoded on the same strand (GP-SD; directional). To study domain colocalization a nearest neighbor approach was applied using a sliding window of 20 protein domains**.** Table 1 summarizes the results obtained. A total of 438 GPs were not present in any of the investigated *Pseudomonas* strains. The majority of these GPs represent functions and processes typically found in eukaryotic species (Supplementary Table S3). Conversely, using the GP-PA method, a functional GP core of 154 complete GPs was present in all strains. When domain colocalization was used as an additional constraint a functional core of 37 complete, likely operonic, GPs was found with both domain colocalization methods. Note that overall, the GP-SND and GP-SD generated very similar output underpinning a strong linkage between operonic structures and functional genome properties in bacterial species (Table 1). Both approaches require domain colocalization which increases the certainty in annotation of the corresponding GP. We recommend using GP-SND as the annotation method as the results obtained are similar to GP-SD method but does not require strand specific information.

**Table 1:** Average number of strain specific Genome Property classes per approach.

Approach	Complete	Partial	Not detected	Not present^a^
GP-PA	440 ± 22	256 ± 14	590 ± 14	438
GP-SND	161 ± 11	362 ± 6	763 ± 12	596
GP-SD	158 ± 10	365 ± 7	763 ± 13	602

^a^Number of genome properties not presented in any of strains.

**Figure 3:**
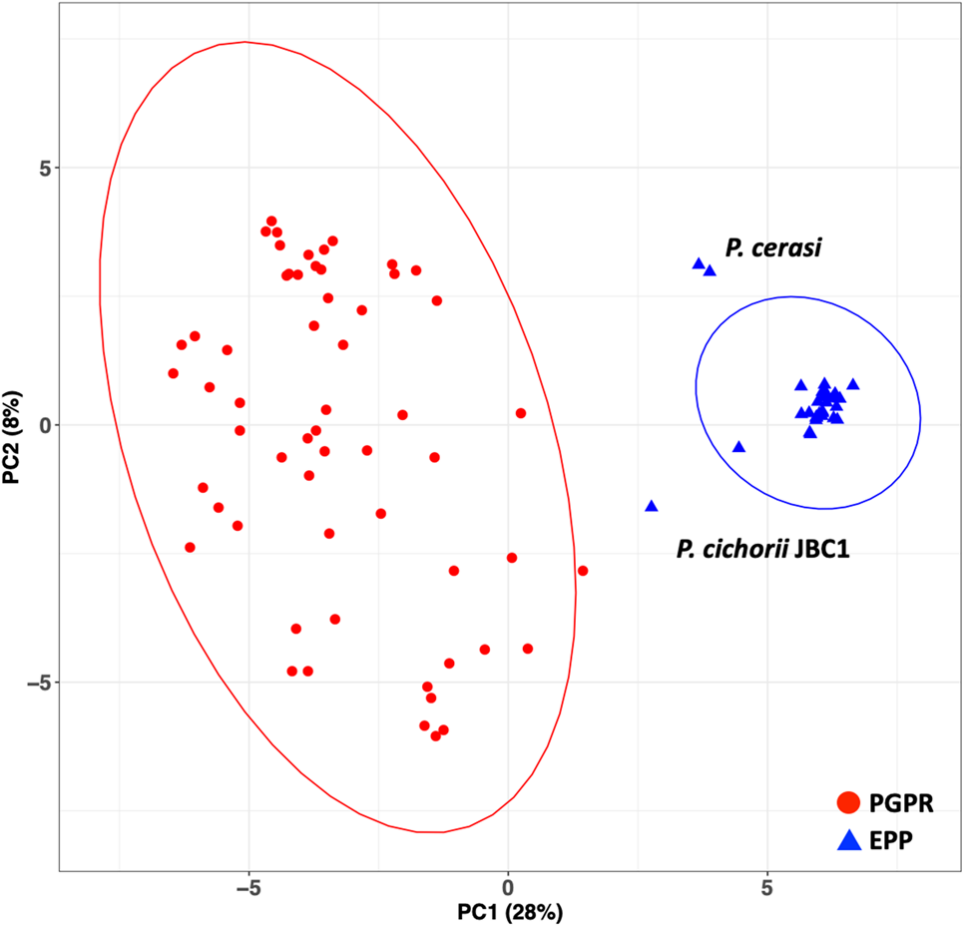
Principal component analysis based on GP-SND content as variables. The fraction of the variance is given in parentheses. *P. cichorii* JBC1 and two strains of *P. cerasi* are outside 95% confidence ellipse of the EPP group.

Next, a principal component analysis (PCA) was applied to the GP data. For all three data sets a clear separation between the two groups were obtained (Supplementary Fig. S1). Figure 3 shows the results obtained with the GP-SND approach. To further understand the contribution of each GP to the separation, we performed an enrichment analysis on the results obtained with the three clustering approaches (Supplementary Table S3). The enrichment analysis was performed on the binary data of presence and absence of the properties by considering “PARTIAL” as presence or absence separately, creating two enriched sets per approach. Subsequently, the two enriched sets were intersected to create the enriched set for that approach. Lastly, an overall enriched set was constructed by considering only the GPs that were enriched in the GP-SD and GP-SND approaches (Table 2).

**Table 2:** Genome properties related to the plant-associated lifestyle: enrichment analysis.

Genome property	Description	Adjusted P-value
**GPs enriched in PGPR strains**		
GenProp0238^a^	2-Aminoethylphosphonate catabolism to acetaldehyde	< 10^–6^
GenProp0721^a^	2-Aminoethylphosphonate (AEP) ABC transporter, type II	< 10^–6^
GenProp0613^a^	Cytochrome c reductase	< 10^–6^
GenProp0907	Poly-beta-1,6 N-acetyl-D-glucosamine system, PgaABCD type	< 10^–6^
GenProp0271	Trehalose utilization	< 10^–6^
GenProp1745	GA12 biosynthesis	< 10^–6^
GenProp1189	MqsRA toxin-antitoxin complex	< 10^–6^
GenProp1645	Zeaxanthin biosynthesis	< 10^–6^
GenProp0659	Tryptophan degradation to anthranilate	7.96 × 10^–5^
GenProp0895	Alcohol ABC transporter, PedABC-type	7.01 × 10^–4^
GenProp0902	Quinohemoprotein amine dehydrogenase	1.40 × 10^–3^
GenProp1516	Phosphatidylcholine biosynthesis V	5.37 × 10^–3^
**GPs enriched in EPP strains**		
GenProp0908^a^	2,3-Diaminopropionic acid biosynthesis	< 10^–6^
GenProp0813^a^	Pyrimidine utilization	< 10^–6^
GenProp1165^a^	PhnGHIJKL complex	< 10^–6^
GenProp1381	Methylphosphonate degradation I	< 10^–6^
GenProp0236	Phosphonates ABC transport	2.62 × 10^–3^
GenProp0710	Generic phosphonates utilization	2.62 × 10^–3^
GenProp1193	RelBE toxin-antitoxin complex	3.19 × 10^–2^
GenProp1566	d-Galactonate degradation	3.64 × 10^–2^

^a^These Genome Properties are also important random forest features (Table 3).

To extend our analysis utilizing the full information of the classes and to capture feature importance, a Random Forest (RF) classifier was built using the annotation results of GP-SND as training-validation set. For 99% of the strains, the RF classifier correctly predicted the lifestyle (EPP or PGPR). The only exception was *Pseudomonas cichorii* JBC1, a causal agent of leaf spot on soybeans but classified by RF-classifier as PGPR. The performance of the RF model was validated using 90% of the data through 100 iterations. First, the ROC curve compared between the best and the worst prediction of the default RF model settings (ntree = 500 and mtry = 20). The AUC shows the identical results of 0.985. Next, we tuned the ntree parameter with the parameter range from 500 to 5000 with 500 steps. The mean of the error rate stabilized at 1.09 ± 0.01% across all number of ntree. However, the variations are lower as the number of ntree increases. Lastly, we tuned the mtry parameter with the parameter range from 1 to 50. The error rate drastically dropped from mtry = 1 to mtry = 2 and stabilized after mtry = 10. The results show the robustness of the default RF settings and indicated that the models are not overfitted (Supplementary Fig. S2).

**Figure 4:**
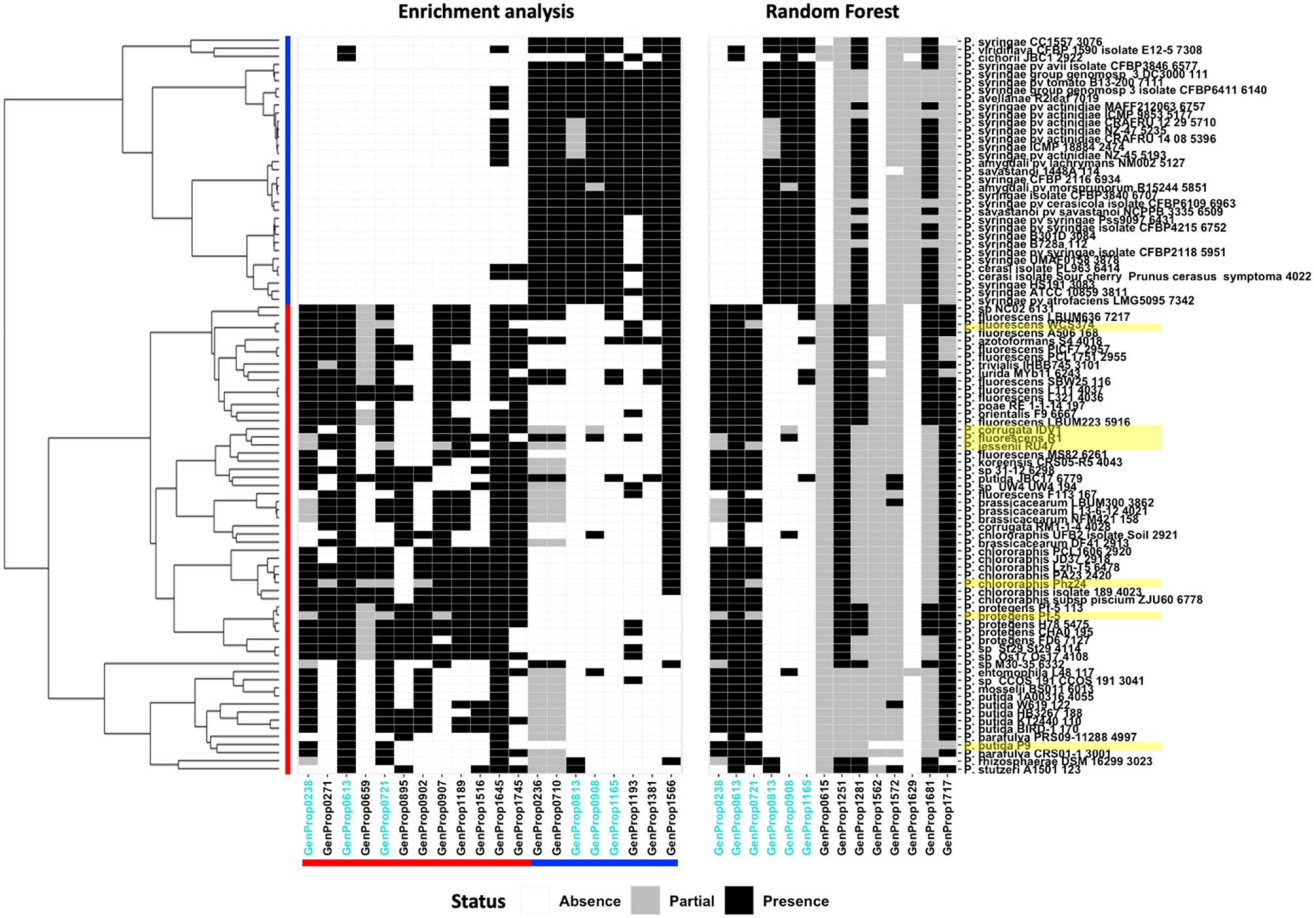
Representative list of discriminating Genome Properties obtained with the GP-SND approach. Left panel: enrichment analysis, right panel: Random Forest feature selection. Red lines indicate the PGPR strains (vertical) and enriched traits (horizontal). Blue lines indicate the EPP strains (vertical) and enriched traits (horizontal). Newly sequenced strains are highlighted in yellow. Enriched GPs that were also highlighted in the RF feature importance analysis are indicated in green.

To study the discriminating variables further, variable selection from RF was implemented (Table 3 and Supplementary Table S3). These variables were integrated with the list of enriched GPs to generate a comprehensive list of key genomic features associated with the plant-associated lifestyle (Fig. 4). A total of 28 variable GPs (Tables 2 and 3) were selected as the discriminating features by the combination of methods. Subsequently, the predictive power of the selection was re-validated by training a RF classifier with only these features. The classification results were consistent with the previously observed groupings.

**Table 3:** Random Forest features importance of Genome properties related to the plant-associated lifestyle.

Genome property	Description	Predictive power^b^
GenProp0813^a^	Pyrimidine utilization	500
GenProp0908^a^	2,3-Diaminopropionic acid biosynthesis	500
GenProp0721^a^	2-Aminoethylphosphonate (AEP) ABC transporter, type II	329
GenProp0238^a^	2-Aminoethylphosphonate catabolism to acetaldehyde	328
GenProp0615	Cytochrome c based oxygen reduction and quinone re-oxidation	251
GenProp0613^a^	Cytochrome c reductase	243
GenProp1629	Propanoyl-CoA degradation I	215
GenProp1572	l-Carnitine degradation I	145
GenProp1562	Fatty acid salvage	53
GenProp1717	Fatty acid beta-oxidationI(GenProp1308, GenProp1510 and GenProp1544)	53
GenProp1165^a^	PhnGHIJKL complex	2
GenProp1251	l-Tyrosine biosynthesis I	2
GenProp1281	Hydrogen sulfide biosynthesis I	1
GenProp1681	l-Cysteine degradation III	1

^a^GP also found in the enrichment analysis.

^b^Numbers were obtained using recursive feature elimination (500 iterations).

### Prediction validation

**Figure 5:**
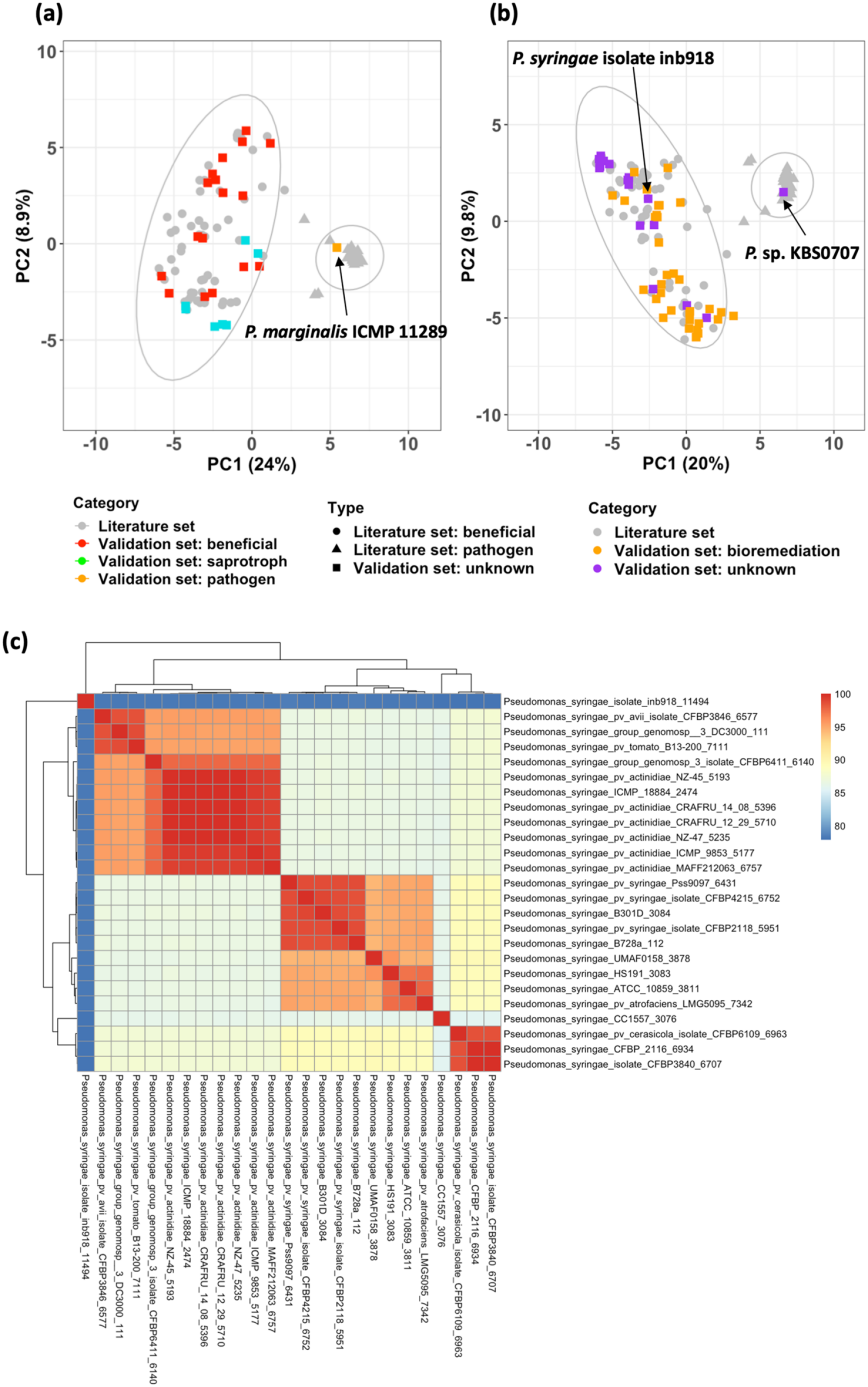
Analysis of the validation set. (**a**) Principal component analysis of the test set 1 composed of PGPR strains (red squares), saprotroph strains (green squares), and EPP (orange square). (**b**) Principal component analysis of the test set 2 composed of bioremediation strains (orange squares) and unclassified strains (purple squares). Variance is indicated in brackets. Previously analyzed *Pseudomonas* strains and previous obtained 95% confidence ellipses are in gray. (**c**) Average Nucleotide Identity (ANI) score among *P. syringae* strains. *P*. *syringae* isolate inb918 is at the top left.

Two test sets of newly retrieved *Pseudomonas* genome sequences were analyzed for the presence of GPs using the GP-SND approach and used in RF performance evaluation (Supplementary Table S1). The first test set consisted of 25 new strains and was a combination of known beneficial and saprobic strains and a strong pathogen. The results confirmed the capability of GP content to predict the plant-associated lifestyle. A PCA of the full dataset (training-validation and test set1) indicated that the separation between two lifestyles was retained (Fig. 5a). Furthermore, we were able to distinguish the strong pathogenic *P. marginalis* ICMP 11,289, recently reclassified as a *P. viridiflava* strain^29^ from the other *P. marginalis* strains which were classified as saprotrophic strains (Fig. 5a)^29^. The second set of 50 strains set was composed of phenotypically unclassified and bioremediation strains. We observed clustering of bioremediation and known PGPR strains (Fig. 5b). Unclassified strain *Pseudomonas* sp. KBS0707 was positioned within the EPP group. As all *P. syringae* are considered to be EPP, the unclassified *P. syringae* isolate inb918 was of interest as it appeared to be a plant beneficial strain. The ANI score suggests that strain inb918 might have been taxonomically misclassified as among the *P. syringae* strains the pair-wise score between this strain and the others remained below 79% (Fig. 5c). Lastly, the RF classifier was applied to the test set yielding the same predictions as the PCA.

## Discussion

Plants live in symbiotic interactions with microbial communities, which are complex networks of interacting nodes. The sum of these interactions can be beneficial for plant growth and development, detrimental or neutral. Many important plant growths promoting bacteria as well as plant pathogens belong to the genus *Pseudomonas*. The genomic diversity observed at species and strain level suggests that *Pseudomonas* spp. have a broad potential for evolutionary adaptation to different environments. Consequently, the plant-associated lifestyle of a *Pseudomonas* strain is likely to be the result of a combinatorial accumulation and emergence of a diverse set of contributing traits.

Differences between PGPR and EPP strains emerged at all levels of analysis. At genome sequence similarity level, a separation between the two groups was prominent. As most of the described phytopathogenic genomes in the scientific literature are obtained from *P. syringae* strains isolated from above ground plant tissue, a high degree of sequence similarity was observed within the EPP group. The ANI score, however, does not consider strain-specific genetic diversity observed within many bacterial species. Strain level diversity has been studied with machine learning techniques for the identification of novel bacterial virulence factors at both DNA and protein domain level^25,30^. In this study machine learning was applied to identify genome wide functional differences between *Pseudomonas* PGPR and EPP strains.

The main limitation of this study is the lack of phenotypic information. To describe the differences between the lifestyles, the strain specific phenotypic information required need to be as complete as possible but available phenotypic data is often unbalanced and hidden in multiple unstructured textual literature sources, seriously hampering information accessibility. In addition, we screened for plant associated *Pseudomonas* strains with a ‘complete’ genome. As a result, the strains selected by these criteria were isolated from two main locations PGPR from soil, and EPP from above ground plant tissues. The functional differences observed in this study are therefore assumed to be derived from both environmental adaptations and virulence factors. Decoupling these factors is difficult as many virulence factors primarily serve general adaptation purposes, and it is their association that promote pathogenesis of susceptible hosts. In addition, strains of *P. syringae* have also been isolated from soil, water, and snow^31,32^.

By focusing on the reconstruction of domain-based GPs, random forest feature independence is promoted, and the complexity of the RF-model is reduced. In total 848 different domain-based GPs were annotated to be (likely) present in one or more of the here studied *Pseudomonas* strain. Underpinning the genomic diversity of the *Pseudomonas* spp. used in this study, in contrast a functional core of only 154 complete and persistently present GPs was obtained. While for obvious reasons by far most of the typical eukaryotic GPs were not detected, a limited number of the *Pseudomonas* GPs have domain overlap with GPs of similar function typically found in eukaryotic species. An example is the domain overlap between GenProp1717 and the “peroxisomal” GPs GenProp1308, GenProp1510 and GenProp1544 all involved in fatty acid beta-oxidation which we treated as one.

Three different approaches were used to determine the domain-based GP content of each strain. Implementation of domain colocalization as a constraint mirrors the operonic structures common in bacterial genomes^33^. For the domain colocalization methods a sliding window of 20 domains was chosen as it would covers 1255 of the 1286 GPs (98%) with the most abundant group of GPs being GPs requiring two evidences (396 GPs) (Supplementary Fig. S3). The average copy number of a single domain is 2.3, indicating that the same domain could be assigned to multiple functions across the genome. Inclusion of protein domain colocalization in GP reconstruction therefore also increases the prediction certainty of those GPs and further promotes the selection of accessory traits, some of which may be acquired by lateral transfer, as RF-variables in RF training. Very similar results were obtained with GP-SND and the strain specific GP-SD method, suggesting that domain clustering most likely exposes operonic structures.

As various *Pseudomonas* species in our list are represented by both pathogenic and non-pathogenic strains, we assumed that the variable genomic regions contributing to these phenotypes will also be variably present between strains of these species. Other variable regions may be important for the specific growth environment (soil or epiphytic) or due to phylogenetic differences between the various groups. To capture the functions encoded by such variable genomic regions we specifically focused on operonic GPs with all required evidences clustered within a defined genomic region. We assumed that a number of the variable operonic functions would correlate with the plant-associated lifestyle (EPP or PGPR). Overall, we detected a common core of only 37 operonic GPs and a set of more than 640 variable operonic GPs (Table 1). Initial analysis showed that none of these variable operonic GPs can single handedly be used to separate between the two groups. Subsequently, we used a RF classifier to identify within this large pool of variable GPs discriminating features that may contribute to the plant-associated lifestyle.

To explore the performance of the RF classifier, 75 new soil derived *Pseudomonas* genomes were selected for testing. For most, the RF classifier firmly supported the discrimination between the beneficial and the pathogenic strains. *P. cichorii* JBC1 was classified as non-pathogenic. However, that does not directly translate into it being beneficial. Figure 4 shows that *P. cichorii* JBC1 still contains three GPs associated with pathogenicity: ‘2,3-diaminopropionic acid biosynthesis’ (GenProp0908), ‘RelBE toxin-antitoxin complex’ (GenProp1193) and ‘D-galactonate degradation’ (GenProp1566). *P. cichorii* JBC1 has already been reported to be quite different to other pathogenic *Pseudomonas* at the genome level^34^ and our results confirm this finding suggesting that there may be other mechanisms for pathogenicity associated with this strain.

RF recursive feature elimination and GP enrichment analysis was used to select a minimal set of GP-variables needed for a good prediction of the predefined plant-associated lifestyle (PGPR or EPP). GenProp0238 and GenProp0721 are two of those important RF-variables (Table 3) and are shown to be enriched in PGPR strains (Table 2). The two GPs are related to mechanisms of phosphonate utilization, which have been shown to occur in *Pseudomonas* and also in other microorganisms^35^. Phosphonate is a form of phosphorus, which is essential for biological processes, for example the synthesis of nucleic acids and phospholipids^36^. However, both groups show differences in the usable form of phosphonate. Most PGPR strains appear to be able to utilize only 2-aminoethylphosphonate (AEP) via the genome properties: ‘2-aminoethylphosphonate catabolism to acetaldehyde’ (GenProp0238) and ‘2-aminoethylphosphonate (AEP) ABC transporter, type II’ (GenProp0721), whereas the EPP strains appear to be able to access broader forms of phosphonates, as also shown by the enriched protein domain, via ‘phosphonates ABC transport’ (GenProp0236), ‘generic phosphonates utilization’ (GenProp0710), ‘PhnGHIJKL complex’ (GenProp1165) and ‘methylphosphonate degradation I’ (GenProp1381)^37^. AEP is the most abundant C-P compound in nature while other phosphonates and their derivatives are substances used in agriculture (herbicides, fungicides and insecticides) and pharmacy (antibiotics)^38^. It has been reported that the virulence of pathogenic species was enhanced under conditions of orthophosphate limitation^39^. Thus, we hypothesize this could be due to the presence of genome traits that enable them to access a wider set of phosphate sources.

GenProp0908 is another important RF-variable. This GP was found to be enriched in EPP strains and is involved in 2,3-diaminopropionic acid biosynthesis (DAP). DAP is a precursor of several secondary metabolites, such as siderophores, neurotoxins and antibiotics^40^. Pyoverdine, the principal siderophores, from the beneficial *P. fluorescens* C7R12 have been reported to reduce Arabidopsis immunity in exchange with the growth under iron deficiency condition^41^. The vulnerability caused may be one of the offense mechanism for other pyoverdine producing pathogenic *Pseudomonas*, such as *P. syringae* and *P. cichorii*^42^. Siderophores are important metabolites involved in iron acquisition^43^. Iron is crucial to many metabolic processes and is therefore required to maintain cells in a healthy state^44^. The stronger ability to scavenge for iron, and the phosphonate previously mentioned, will increase the fitness of the pathogens.

Two GPs strongly enriched among the PGPR strains are GenProp0907, and GenProp0902 (Table 2). GenProp0907 represents a cluster of four genes involved in the synthesis, modification and export of the biofilm adhesin poly-beta-1,6-*N*-acetyl-d-glucosamine and the four domain evidences represent the four genes required. The GP is not present in the EPP group and found to be complete as likely operonic structures in 39 PGPR strains. Biofilms of the PgaABCD type have been studied in *Escherichia coli*^45^ but not in *Pseudomonas* species. GenProp0902 represents quinohemoprotein amine dehydrogenase (QHNDH). QHNDH is a three-subunit enzyme located in the periplasmic space of *P. putida* and part of the amine oxidation respiratory chain. QHNDH catalyzes the oxidative deamination of primary amines when used as a sole carbon and energy source^46^. The GP consists of four evidences, three domains representing the alpha-, beta- and gamma-subunit of the enzyme and one representing the QHNDH maturation protein. This likely operonic GP was found to be complete in 24 biocontrol strains and is not present in the EPP group. As these GPs are only present in subset of the PGPR strains, they did not emerge as important RF-variables in recursive feature elimination.

Protein domains associated with Type II secretion system (T2SS) were found to be enriched among the PGPR strains while domains involved in the type III secretion system (T3SS) were found to be enriched among the EPP strains. T2SS is captured by GenProp0053 and consists of 10 non-optional evidences and 3 optional domains. GP results however, indicated for both groups a “PARTIAL” status for this GP. Similarly, the type III secretion system, represented by GenProp0052 is considered to be a key virulence factor and has been considered as evidence for pathogenicity in many genome studies^19,47,48^. GenProp0052 is a complex GP consisting of 14 evidences and 28 optional domains. Due to the set zero threshold for “PARTIAL” for this specific GP, a single evidence domain will already result in a “PARTIAL” status. Eighteen protein domains enriched in EPP are described to be involved in Type III secretion systems. Eleven of those enriched domains are used as evidences for GenProp0052. One other, TIGR02551, did also occur in the pathogen set but was considered not to be enriched after the Bonferroni adjustment. In contrast, the two missing evidences, TIGR02105 and TIGR02546 are only present in five PGPR genomes. Thus, amongst the tested 91 *Pseudomonas* strains all of the 14 required evidences are present, but none of the strains used in this study have the complete set.

Due to the ‘Partial’ status of GenProp0053 (T2SS) and GenProp0052 (T3SS) for both lifestyles these GPs were not enriched, nor were they selected as discriminating variables in RF classification. We further examined the distribution of the GenProp0053 and of GenProp0052 evidences over all strains (Supplementary Fig. S4). The distribution showed that protein domains linked to GenProp0052 more consistently occurred in the EPP group with more variation in the PGPR group. The result suggests that the abundance of T3SS related domain content could be sufficient for an indication of the pathogenicity. However, due to the missing evidences, there is no guarantee that the feature is functional. Moreover, *P. syringae* naturally lacking the canonical T3SS can still be pathogenic^49,50^, while some strains contain multiple T3SSs of which the role is still unknown^51^.

Specifically, for the PGPR group a number of enriched GPs suggested a role for pathways involved in the degradation and utilization of trehalose (GenProp0271), tryptophan (GenProp0659) (Table 2), tyrosine (GenProp1251) and carnitine (GenProp1572) (Table 3). On the other hand, EPP strains appears to be more specialized in the degradation of galactonate (GenProp1566) and cysteine (GenProp1681). Carbon sources that were predicted to be degradable by preferably the PGPR group could contribute to the agricultural industry. These substrates could be used as fertilizers, growth promotors, or as additives to alternate the microbial composition^52^. Similar to elicitors, which directly enhance plant defense and resistance, this indirect approach could be applied to the existing microbial community to select for the beneficial strains and potentially increase the productivity of the crop^53^. On the other hand, carbon sources that might prolong saprobic growth and survival of pathogens should be avoided.

Other GPs found in the PGPR group are linked to four ‘human hormones’, which are ‘mineralocorticoid biosynthesis’ (GenProp1644), ‘estradiol biosynthesis II’ (GenProp1417), ‘glucocorticoid biosynthesis’ (GenProp1666) and ‘pregnenolone biosynthesis’ (GenProp1740). The evidence shared by these hormones, domain PF00067 (cytochrome P450), is the same as for ‘GA12 biosynthesis’ (GenProp1745). Hence, only GA will be further discussed. Gibberellin 12 (GA_12_), is the common precursor of all gibberellins (GA)^54^. GA phytohormones play important roles in influencing the growth and development of the host plants^55^ and GA from *Pseudomonas* could increase seed germination^56^.

Not all known virulence traits are represented by a GP. Many of those are found in plant pathogens such as, coronatine, cytokinin and auxin, conserved effector locus (CEL) and exchangeable effector locus (EEL)^57–59^. We examined the presence of the protein domains associated to these traits in our dataset (Supplementary Fig. S5). The results showed that the associated protein domains are generally present in both groups. Among these domains, only PF08659 and PF16197 were enriched in the EPP group. This suggests that the occurrence of these, known to be, plant pathogenic traits may not be sufficient as a genetic marker to identify the pathogenicity of a strain.

In conclusion, domain-based Genome Properties appear to be robust computational features to differentiate between PGPR and EPP *Pseudomonas* strains and our analysis shows that incorporation of domain colocation further increases their relevance. By combining traditional statistical analysis (enrichment analysis) and machine learning methods (random forest) we were able to identify new discriminating genome properties that can be used to identify species that promote plant growth. These could be applied in strategies to develop synthetic PGPR communities and to formulate soil additives to improve plant health and performance.

## Methods

### Genome retrieval and annotation

*Pseudomonas* genomes were downloaded from Pseudomonas Genome DB version 17.2. The test set was obtained from database version 20.2 (https://www.pseudomonas.com)^27^. Genomes were manually categorized according to their lifestyles using literature data (Supplementary Table S1). Additionally, 7 genome sequences were (re)sequenced from phytobeneficial strains *P. putida* P9 (accession ERS6670306), *P. Corrugata* IDV1 (accession ERS6652532), *P. fluorescens* R1 (accession ERS6670181), *P. protegens* Pf-5 (accession ERS6652530), *P. chlororaphis* Phz24 (accession ERS6670416), *P. jessenii* RU47 (accession ERS6670307) and *P. fluorescens* WCS374 (accession ERS6652531). DNA was extracted using the Epicenter Masterpure kit (Epicentre Technologies, USA) according to the manufacturer’s protocol and quantified with the Infinite® 200 PRO (Tecan, Männedorf, Switzerland) using the Quant-iT™ PicoGreen™ dsDNA Assay Kit (ThermoFisher, Waltham, USA) according to the manufacturer’s protocol. The strains were sequenced on the PacBio Platform (Pacific BioSciences, Menlo Park, USA). A total of 4 µg DNA was sheared to 7 Kb and two SMRT bell libraries were prepared using the kit Barcoded Adapters for Multiplex SMRT sequencing in combination with the Sequel Binding Kit V2.0 and the Sequel Polymerase 2.0 Kit. Per library, a pool with sheared DNA of all strains was used as input according to the manufacturer’s protocol. Sequencing was done on a Sequel system operated at the services of Business Unit Bioscience, Wageningen Plant Research (Wageningen, The Netherlands). Subsequently, de-multiplexing was performed by aligning the barcodes to the sub-reads with pyPaSWAS version 3.0^60^. Canu version 1.6^61^ was used to assemble the PacBio reads.

The SAPP semantic annotation framework^62^ was used to systematically (re)annotated the genomes. Briefly, protein encoding genes were de novo predicted using Prodigal 2.6.3^63^ using the gene caller.jar module with the following arguments: -prodigal and -codon 11. The protein domains were characterized with InterProScan 5.36–75.0 using the Pfam and TIGRFAMs databases^64–66^ using the InterProScan.jar module with the following arguments: -a PFAM,TIGRFAM. Annotation data and meta-data was stored in a semantic database using the GBOL ontology^67,68^. SPARQL queries were used to extract protein domain identifiers, and the location and direction of the corresponding gene.

### Data processing

OrthoANI version 1.40 was used to calculate the Average Nucleotide Identity (ANI) score for all genomes^69^. PygenProp, was used to infer from each genome domain-based GPs^70^. Three criteria were applied; “PA”, considering only domain presence as evidence, “SND”, synteny-non-directional, requiring the genome location of the corresponding domains to be in close proximity and “SD” that in addition to gene location also considers strandness. For SND and SD a nearest neighbor approach and a sliding window of 20 protein domains was applied. Each GP was classified as either ‘YES’, or ‘PARTIAL’ according to the completeness of the set of evidences.

### Statistical analysis

The natural grouping of the data was visualized using principal component analysis (prcomp package). Then, with R packages; fisher.test and p.adjust, Fisher Exact Test with Bonferroni correction was applied to protein domains and the genome properties to test for enrichment. This analysis identified the over- and under-represented features. GP data was reassessed twice by considering ‘PARTIAL’ as either ‘YES’ or ‘NO’. The enriched list was created by intersecting the two cases of ‘PARTIAL’. Enrichments were considered significant if the adjusted p-value after Bonferroni correction of the GP is below 0.05.

The Random Forest classifier was created using R package randomForest v4.6-14^71^ using the default settings. Labelled data were divided into training, validation, and test sets. The training-validation set was used to access the performance of the model using 90% of the data with 100 iterations. The performances were measured using the ROC curve of the default parameters and parameters tuning with both ntree and mtry respectively. A tenfold cross validation was used. The unbiased training set was created with equal numbers per group determined by using 75% of the smaller group, the EPP group, resulting in 25 strains chosen at random per group. Therefore, the validation set remains with 33 PGPRs and 8 EPPs. The Variable Selection from Random Forests v 0.7-8 (varSelRF) package in R was used to determine variable importance. We used 5000 trees for the first forest and 2000 trees for all additional forests during the iteration. Vars.drop.frac, the portion of the variable that is excluded on each iteration, was set to 0.2. For testing purposes two sets of strains were used, one was composed of 17 PGPR strains, 7 saprotrophic strains and 1 plant pathogen. The second set was composed of 34 bioremediation strains and 16 unclassified strains.

## Supplementary Information

Supplementary Figure S1.

Supplementary Figure S2.

Supplementary Figure S3.

Supplementary Figure S4.

Supplementary Figure S5.

Supplementary Table S1.

Supplementary Table S2.

Supplementary Table S3.

## Data Availability

The input files and code are available at: https://gitlab.com/wurssb/pseudomonas-genome-properties.
