# Supplementary material for: Classification of the plant-associated lifestyle of Pseudomonas strains using genome properties and machine learning
Source: Sci Rep. 2022 Jun 27;12:10857. doi: 10.1038/s41598-022-14913-4 (PMC9237127; doi:10.1038/s41598-022-14913-4)
Supplement: Supplementary file 1 — Supplementary Figure S1. [file 41598_2022_14913_MOESM1_ESM.docx]

1. **(b) (c)**

***P. cichorii* JBC1**

***P. cichorii* JBC1**

***P. cerasi***

***P. cerasi***

***P. cerasi***

***P. cichorii* JBC1**

**PGPR EPP**

**Supplementary Figure S1: PCA based on the GPs constructed using 3 approaches.** (a) GP-PA, (b) GP-SD and (c) GP-SND (Figure 3). The fraction of the variance is given in the parentheses. The PCA retained the separation between the groups. Differences between two *P. cerasi* could be observed using the latter two approaches.
