# Supplementary material for: Classification of the plant-associated lifestyle of Pseudomonas strains using genome properties and machine learning
Source: Sci Rep. 2022 Jun 27;12:10857. doi: 10.1038/s41598-022-14913-4 (PMC9237127; doi:10.1038/s41598-022-14913-4)
Supplement: Supplementary file 2 — Supplementary Figure S2. [file 41598_2022_14913_MOESM2_ESM.docx]

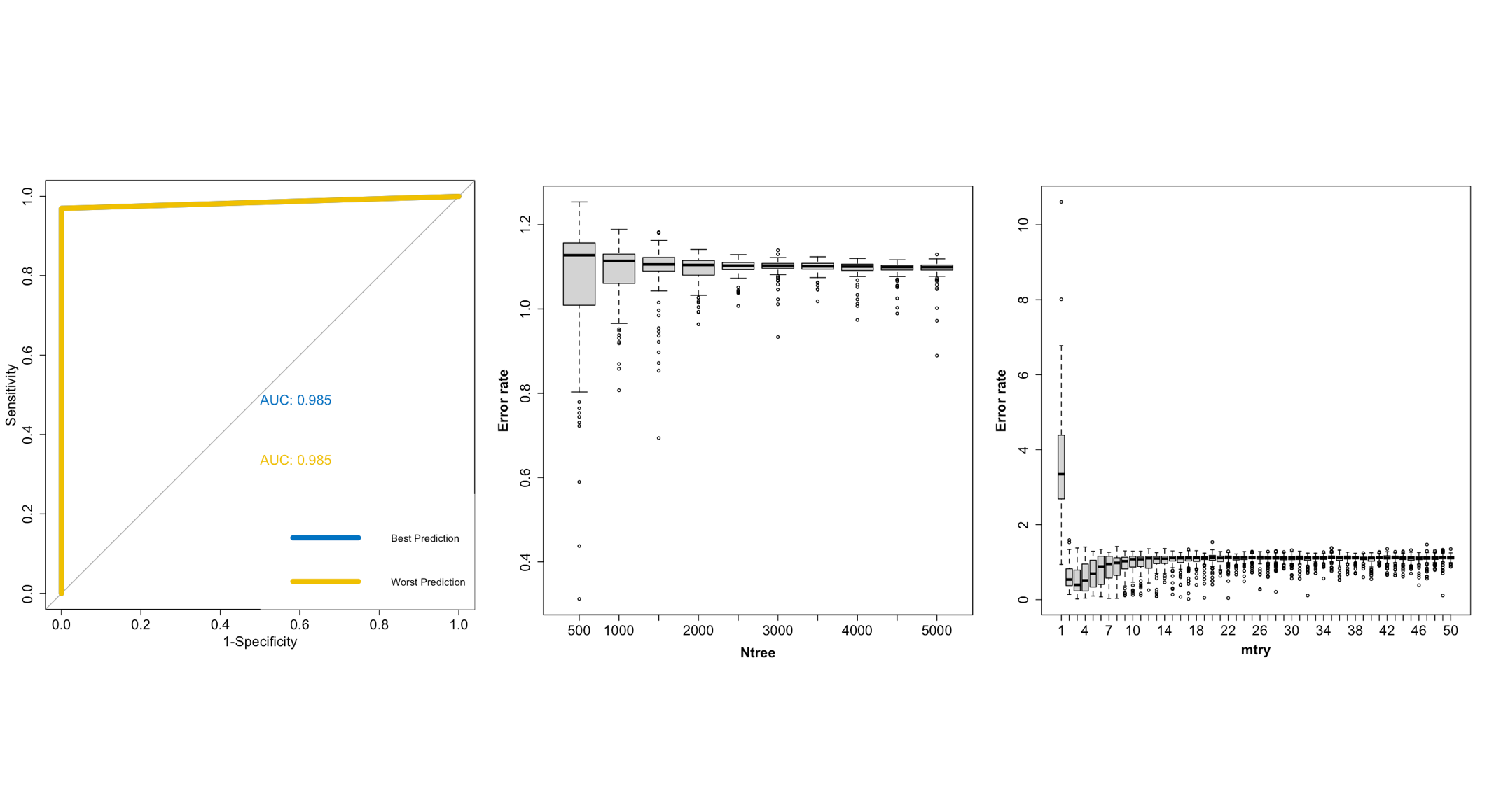


1. **(b) (c)**

**Supplementary Figure S2: Validation of RF model performance.** (a) An ROC curve of the RF model showing identical best and worst performances, (b) Error rate of the RF model from the ntree parameter tuning of 100 iterations with the parameter range from 500 to 5000 with steps of 500, and (c) Error rate of the RF model from the mtry parameter tuning of 100 iterations with the parameter range from 1 to 50.
