# Supplementary material for: Classification of the plant-associated lifestyle of Pseudomonas strains using genome properties and machine learning
Source: Sci Rep. 2022 Jun 27;12:10857. doi: 10.1038/s41598-022-14913-4 (PMC9237127; doi:10.1038/s41598-022-14913-4)
Supplement: Supplementary file 3 — Supplementary Figure S3. [file 41598_2022_14913_MOESM3_ESM.docx]

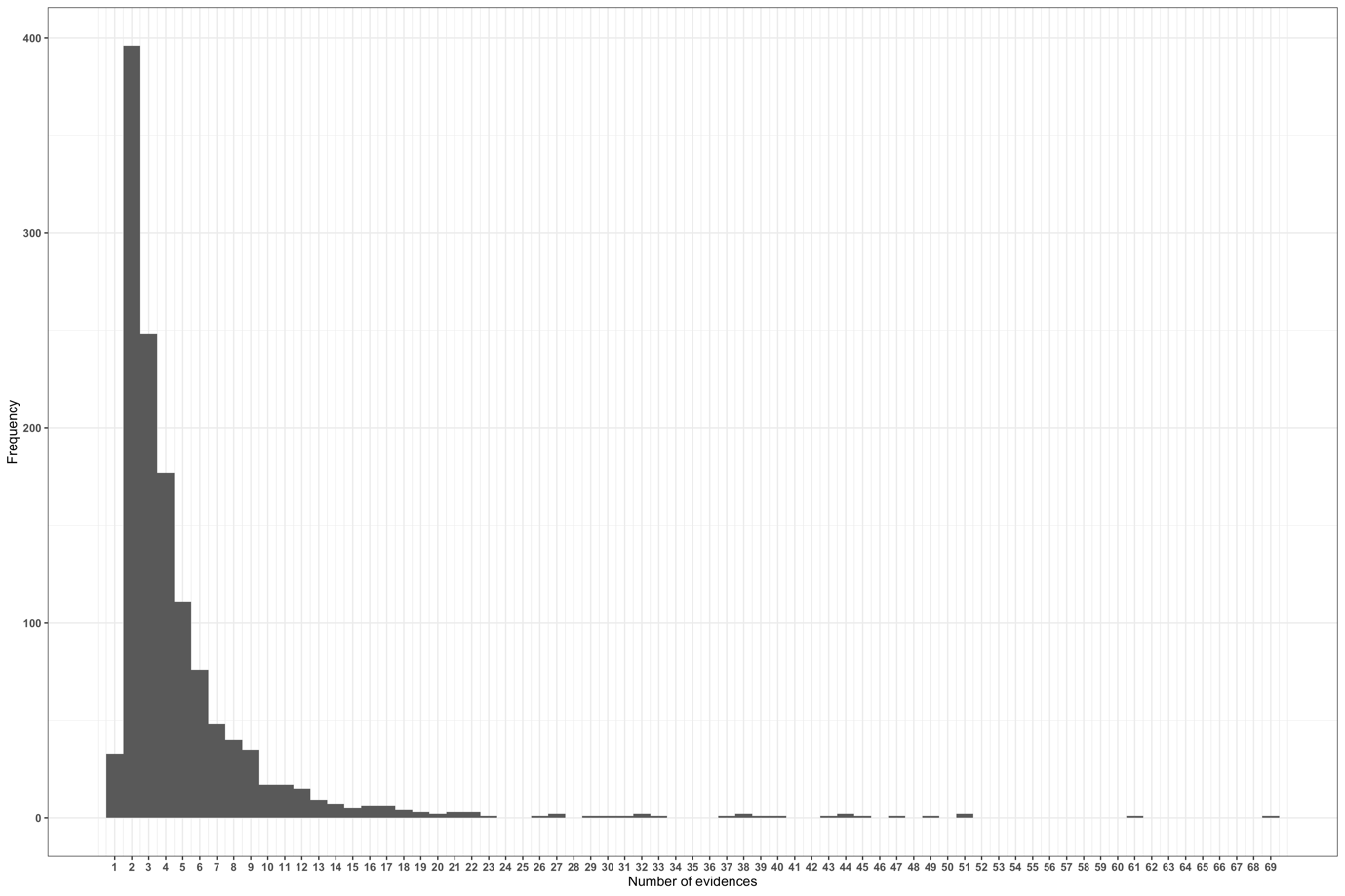


**Supplementary Figure S3: Distribution of number of evidences occurred in the Genome Properties.**
