# Supplementary material for: Classification of the plant-associated lifestyle of Pseudomonas strains using genome properties and machine learning
Source: Sci Rep. 2022 Jun 27;12:10857. doi: 10.1038/s41598-022-14913-4 (PMC9237127; doi:10.1038/s41598-022-14913-4)
Supplement: Supplementary file 4 — Supplementary Figure S4. [file 41598_2022_14913_MOESM4_ESM.docx]

**PGPR**

**EPP**

**Supplementary Figure S4: Distribution of the number of non-optional evidence for (a) GenProp0053 (Type II secretion systems) and (b) GenProp0052 (Type III secretion systems).** Fraction of strains are on top, and the number of strains is represented in the bracket.
