# Supplementary material for: Classification of the plant-associated lifestyle of Pseudomonas strains using genome properties and machine learning
Source: Sci Rep. 2022 Jun 27;12:10857. doi: 10.1038/s41598-022-14913-4 (PMC9237127; doi:10.1038/s41598-022-14913-4)
Supplement: Supplementary file 5 — Supplementary Figure S5. [file 41598_2022_14913_MOESM5_ESM.docx]

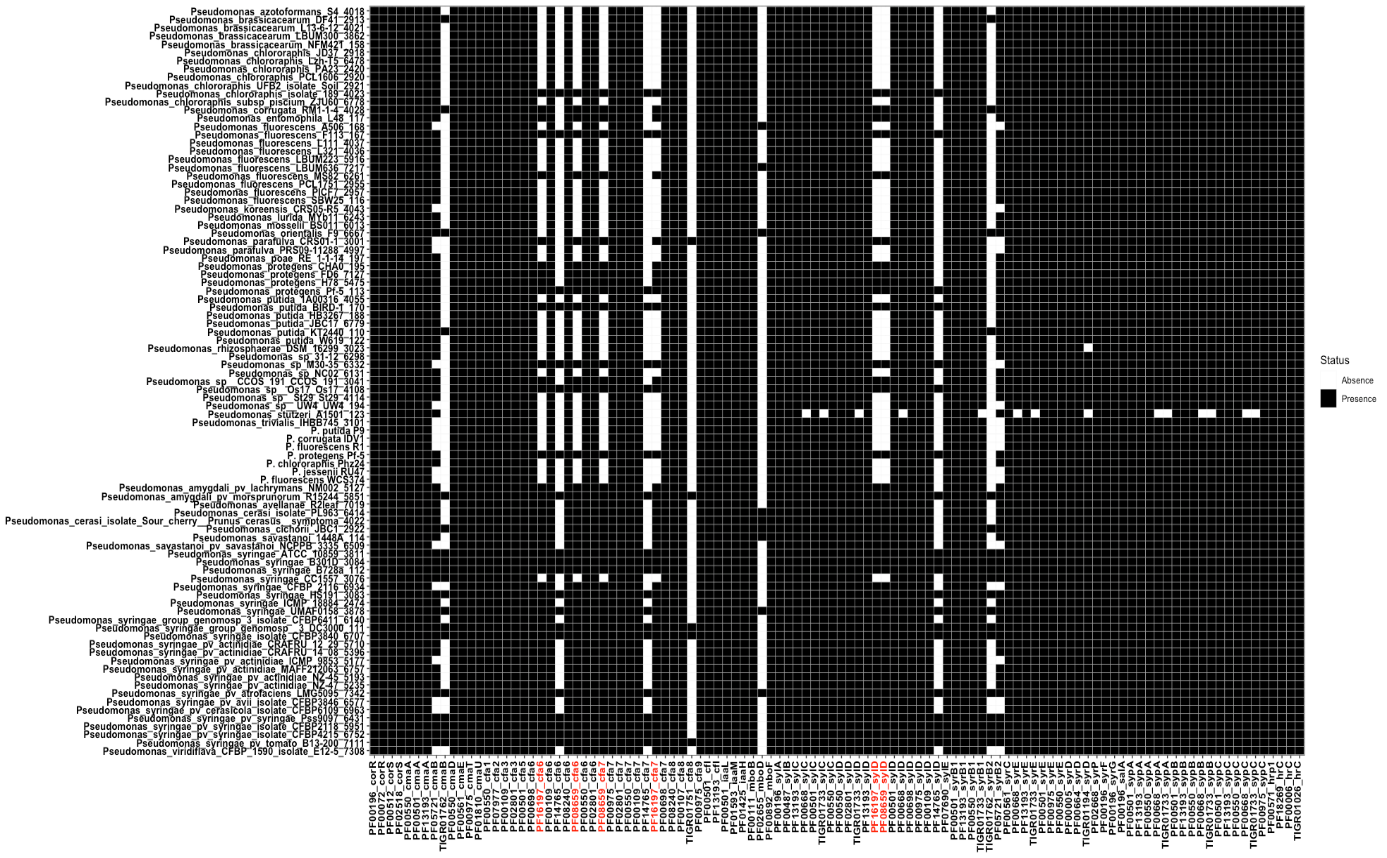


**T3SS**

**Mangotoxin**

**IAA conjugation**

**Coronatine**

**Syringopeptin**

**Syringomycin**

**Syringolin**

**IAA synthesis**

**Supplementary Figure S5: Presence and absence of protein domains associated to genes related to selected pathogenic traits found in *P. syringae***^1^. Colored vertical line distinguished between the beneficial (red) and the pathogen (blue) group. Protein domains in red are the member of the enriched protein domains for pathogenic group.

## References

1. Ruinelli, M., Blom, J., Smits, T. H. M. & Pothier, J. F. Comparative genomics and pathogenicity potential of members of the Pseudomonas syringae species complex on Prunus spp. *BMC Genomics* **20**, 172 (2019).
